# Recent ENSO evolution and its real-time prediction challenges

**DOI:** 10.1093/nsr/nwac052

**Published:** 2022-03-23

**Authors:** Rong-Hua Zhang, Chuan Gao, Licheng Feng

**Affiliations:** Institute of Oceanology, and Center for Ocean Mega-Science, Chinese Academy of Sciences, China; Pilot National Laboratory for Marine Science and Technology, China; Institute of Oceanology, and Center for Ocean Mega-Science, Chinese Academy of Sciences, China; Pilot National Laboratory for Marine Science and Technology, China; National Marine Environmental Forecasting Center, Ministry of Natural Resources, China

## Abstract

The frequent occurrences of the second-year surface cooling condition in the eastern equatorial Pacific, as observed in late 2021, are attributed to decadal changes in the thermocline depth, which determine the relative dominances of local cooling effect in the east and subsurface warming effect remotely from the west. Coupled models need to adequately represent these processes in a balanced way, thus being able to successfully predict the observed sea surface temperature evolution in late 2021.

The El Niño-Southern Oscillation (ENSO) is a major interannually reoccurring mode of Earth's climate system that originates naturally from ocean–atmosphere interactions in the tropical Pacific and can affect the weather and climate worldwide. ENSO has been observed to undergo decadal variations and modulations [1–3], including the intensity, asymmetry of its two phases (El Niño and La Niña), different types of El Niño and multiyear La Niña and El Niño events. One well-known example occurred during the so-called 1976–1977 climate shift [4], with El Niño events emerging frequently in the tropical Pacific during the 1980s and 1990s.

Another apparent shift occurred in the late 1990s [5], as indicated by the sea surface temperature (SST) on the equator (Fig. 1a). A corresponding trend includes frequent La Niña events and a fewer El Niño events (Fig. 1a) [6]. For example, La Niña conditions emerged in 2020 in the tropical Pacific. Then, the tropical Pacific was nearly ENSO-neutral in May 2021. Strikingly, cold conditions returned again in late 2021 without sustaining a neutral condition and transiting into warming in the tropical Pacific. Thus, a second-year cooling reemerged in 2021 following the major La Niña condition in 2020 (Fig. 1a), with the turning point occurring in June 2021 (the black line in Fig. 1c representing observed SST anomalies in the Niño 3.4 region). In fact, these consecutive La Niña events occur quite often in this century [5,7]. The evolution of the recent prolonged 2020–2021 La Niña event has been of great interest because this multiyear cold condition is occurring under a warm climate background [2,8]. Currently, the mechanisms responsible for decadal ENSO changes are still not well understood. In particular, a satisfactory explanation for the recent prolonged 2020–2021 La Niña evolution in the tropical Pacific has proven elusive.

Processes causing SST changes in the tropical Pacific include positive and negative feedbacks that operate naturally within the climate system [3]. During a La Niña event, its typical conditions are shown schematically in Fig. 1b [6]. For instance, cold SST anomalies are located in the central-eastern equatorial Pacific; these cold anomalies are accompanied by warm subsurface anomalies in the west, which tend to propagate eastward along the equator. The cold SST conditions in the central-eastern equatorial Pacific are influenced by several processes. Local processes in the eastern equatorial Pacific from the Bjerknes positive feedback act to sustain the cold SST condition. At the same time, the subsurface warming process comes remotely from the western equatorial Pacific in the form of downwelling Kelvin waves, acting to weaken the cold SST anomalies in the east. If the Bjerknes feedback effect can overcome the downwelling Kelvin wave effect, a La Niña is generated. Thus, the future SST evolutions in the east are determined collectively by the local cooling and remote warming effects (Fig. 1b). When the remote warming effects dominate, the SST conditions in the east are more likely to shift to neutral and warming conditions. In contrast, when the local cooling effects dominate, the SST conditions remain cold. Clearly, a competition is at work between positive and negative feedbacks onto SST, which induce a cooling or warming effect, respectively.

The relative dominances of these competing processes in controlling the evolution of SST can be modulated by the thermocline structure and off-equatorial effects at decadal and longer timescales. For instance, the tropical Pacific shifted from the decadal warm phase in the 1980s and 1990s to a decadal cold phase in the 2000s [4,5]. Since the early 2000s, climatic conditions have reversed in the tropical Pacific, where a decadal cold period has prevailed. Correspondingly, the surface trade winds over the tropical Pacific have been strong, and the thermocline in the east is shallow and close to the surface, which acts to produce colder subsurface waters that can more easily influence the SST in the east. As compared with a situation with a deepened thermocline in the 1980s and 1990s, the shoaled thermocline in the eastern equatorial Pacific since the early 2000s brings up colder subsurface waters entrained into the mixed layer; the corresponding large subsurface thermal anomalies can affect SST more strongly. Thus, the changes in the thermocline during this prevailing decadal cold period indicate a preconditioning with which local subsurface cooling effects become stronger, being more likely to sustain a cool SST condition in the eastern equatorial Pacific. According to these arguments, we speculate that during the 2021 SST evolution, the local cooling effects in the east, relative to the warming effects from the west, play a more dominant role in determining

**Figure 1.**
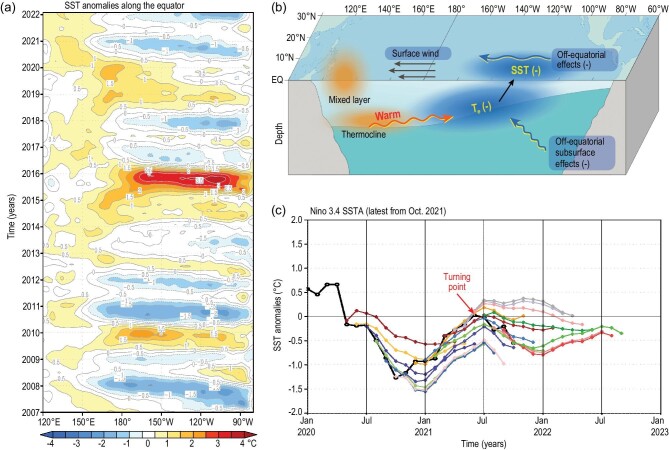
(a) Zonal-time sections along the equator for observed SST anomalies during January 2007–February 2022 from the NOAA OI.v2 SST monthly fields downloaded from the IRI Data Library (the contour interval is 0.5°C). (b) Schematic showing major processes affecting cold SST conditions during 2020–2021 in the central-eastern equatorial Pacific; T_e_ refers to the temperature of subsurface waters entrained into the mixed layer. (c) Niño 3.4 SST anomalies (averaged over the following region: 5°S–5°N; 170°W–120°W) during 2020–2021, as observed (black line) and predicted (colored lines) from the IOCAS ICM; each colored line indicates the trajectory of a 12-month prediction made from different initial conditions. The real-time predictions from various coupled models are shown on the IRI website.

SST evolution in the eastern equatorial Pacific. As a result, cold SST conditions in late 2021 are more likely to be sustained in the east by the stronger cooling effects associated with the temperature of cold waters entrained into the mixed layer, thereby favoring the continuation of the La Niña conditions. This explanation is also applicable to the case for the prolonged La Niña conditions during 2010–2011 [6]. On the other hand, it is obvious that these arguments put a focus on the enhanced cooling effect due to the eastern Pacific shallower thermocline. But what is the role that can be played by the subsurface warming effect from the western Pacific in the resultant cooling SST condition in late 2021? In other words, the sustained cold SST condition in late 2021 could be due to a weakened subsurface warming effect from the western Pacific, and similar arguments can be actually inferred in terms of decadal changes as well. As mentioned above, the decadal cold period over the tropical Pacific since the early 2000s corresponds to strong trade winds and a deep thermocline in the western equatorial Pacific, acting to have a weaker warming effect of subsurface thermal anomalies on the SST, which, in turn, equivalently presents an enhanced cooling effect. So, it is really a subtle competition between positive (cooling) and negative (warming) feedbacks that determine SST evolution in the eastern equatorial Pacific, in which decadal changes can modulate the relative dominances of these cooling and warming effects on SST.

Such notable decadal changes in ENSO evolution can affect the predictability of the ENSO. The ENSO is the most predictable interannual signal in the tropical Pacific and its memory resides in the subsurface ocean [9]. In recent decades, scientific progress in understanding and modeling ENSO processes has reached a stage at which ENSO can be predicted in advance within a range of 6 months to 1 year. For example, an intermediate coupled model developed at the Institute of Oceanology, Chinese Academy of Sciences (IOCAS), named IOCAS ICM, has been routinely used to make real-time predictions [6]; see the summary of ENSO model predictions on the International Research Institute for Climate and Society (IRI) website: http://iri.columbia.edu/climate/ENSO/currentinfo/update.html. Currently, the recognizable ENSO period can be successfully predicted, but it is difficult to predict the ENSO during its transition, say from La Niña conditions to neutral or El Niño conditions, as indicated by the springtime predictability barrier [10]. Therefore, large uncertainties and biases still exist in real-time ENSO prediction. Due to decadal changes in the ENSO, its predictability has also experienced decadal changes. For example, the prediction ability has decreased since the early 2000s; this is in part due to the fact that the precursor of warm water volume in the western Pacific, which provides a lead time of 6–9 months for ENSO, is no longer so since 2000, because many ENSO events since then are central Pacific-type events, which involve less thermocline feedback [7]. Taking 2021 as an example, the June 2021 turning point for the second-year La Niña (Fig. 1c) was difficult to predict using coupled models. In fact, some coupled models failed to predict the occurrence of the second-year cooling in 2021 based on predictions that are made from late 2020 and early 2021 (see the IRI website for details). This failure indicates that these models do not adequately capture the relative dominances of the local cooling and remote warming effects in a balanced way (Fig. 1b). Nevertheless, real-time prediction experiments using the IOCAS ICM indicate that this model can quite successfully capture the SST evolution during 2020–2021 (Fig. 1c). In particular, this model can adequately depict the occurrence of the second-year cooling in 2021, with a turning point in June 2021, when predictions are made in October 2020 (Fig. 1c). As previously explained [6], this model adopts an optimized representation for the temperature of subsurface waters entrained into the mixed layer (T_e_), a field that represents subsurface thermal forcing on SST. T_e_ anomalies in the ICM are determined by its non-local relationship with sea level (SL) anomalies (or equivalently thermocline depth anomalies) in the tropical Pacific, written as T_e_ = α_Te_⋅F(SL), in which F represents interannual T_e_–SL relationships estimated using a singular vector decomposition technique from historical data and α_Te_ is a tunable parameter introduced to represent how T_e_ is affected by thermocline fluctuations and also to quantify the intensity of the T_e_ effects on SSTs. As a result, this model can adequately depict the balanced effects on the SST that are associated with the local and remote processes in the eastern equatorial Pacific. Correspondingly, this model is able to predict the observed SST evolution during 2020–2021 (Fig. 1c), including the second-year cooling in late 2021.

These quantitative arguments are further confirmed by sensitivity experiments using the IOCAS ICM that are designed to illustrate the extent to which different dominances of the competing processes and their effects determine SST evolutions in 2021. As illustrated in [6], the intensity of the local cooling effect (represented by T_e_ anomalies) is critically important to the way in which SST evolves in the eastern equatorial Pacific. We can then use α_Te_ to quantify how the intensity of the T_e_ anomalies affects SSTs. A control prediction run is performed using the IOCAS ICM starting from different initial conditions and taking α_Te_ = 1.0 as its reference value; the results (Fig. 1c) indicate that this model can predict the SST evolution very well, including the turning point in June 2021. Further sensitivity experiments are performed by varying α_Te_. The larger the value of α_Te_ is taken, the more dominant the effect that is represented for the local cooling associated with negative T_e_ anomalies over the remote warming, and thus the stronger the tendency appears for the cold SST anomalies to retain its cold conditions in the east. Particularly, if the local cooling effect is weakly represented below certain levels in the ICM (α_Te_ is set to <0.5), a surface warming can emerge in late 2021. In contrast, when α_Te_ is set to ≥1.0 and thus the cooling intensity of T_e_ is adequately represented, cooling conditions emerge in 2021 (Fig. 1c) and a return of the La Niña conditions in the fall of 2011 is well depicted by the ICM.

The tropical Pacific SST also exhibits a long-term warming trend, indicating that the ENSO can interact with global warming induced by human activities [8]. As researchers have demonstrated, El Niño has warming effects on the global climate, whereas La Niña has an instant global cooling effect, which is usually strongest in the next year of the event. In terms of the relationships between global-warming effects and the ENSO, the latter is likely modulated by the former, but how ENSO will change in the future under a warming climate remains controversial, thereby defining a clear area for future research. Also, improving real-time predictions and future projections of ENSO is clearly needed. Hopefully, mechanism understandings and process representations of ENSO can be transformed into their improved capacities in climate modeling.
